# Mesh-Repaired Complete Sternal Cleft Complicated by Multi-Resistant Bacterial Infection

**Published:** 2014-09-01

**Authors:** Pim B. Olthof, Derk A. Colenbrander, Martijn Van Der Kuip, Hugo A. Heij

**Affiliations:** 1 Emma Children’s Hospital, Academic Medical Center Meibergdreef 9, 1105 AZ Amsterdam, Netherlands; 2 Department of pediatric infectious diseases and immunology, VU University medical center, Amsterdam, Netherlands; 3 Professor of pediatric surgery and head of the Amsterdam pediatric surgical center, Netherlands

**Keywords:** Sternal cleft, Mesh, Infection

## Abstract

Sternal cleft is a very rare congenital anomaly, which can occur as an isolated or associated with other anomalies. We report a patient with a mesh-repaired complete sternal cleft complicated by infection with a multi-resistant Pseudomonas aeruginosa. The patch was surgically removed.

## CASE REPORT

A 16-month-old girl presented with chronically infected midline anterior thoracic surgical scar. History revealed that the child was born in India with absent sternum. CT scan performed at that time also showed complete absence of sternum. Echocardiogram showed situs solitus with an insignificant ventricular septal defect. The sternal defect was bridged surgically using a dacron patch. Operating notes also revealed a pericardial defect found during surgery in the same country. The infection started shortly after surgery, and was treated with mupirocin treatment locally.

On physical examination, there was an infected wound on the lower part of the hypertrophied scar. Further examination revealed a high-arched palate, micrognathia and two small hemangiomas measuring one centimeter in diameter on her left cheek. From the wound a multi-resistant Pseudomonas aeruginosa was cultured, only sensitive to meropenem and colistin. Considering the resistance of the bacteria, the mupirocin was substituted for 1% acetic acid as local treatment. The infection resolved and the wound closed within two months. However, when the acetic acid treatment was discontinued the wound started ulcerating and the same bacteria were cultured again. A thoracic ultrasound showed a fluid collection under the dacron patch, therefore the patch was considered to be colonized with Pseudomonas and a reason for recurrent infections.

Pre-operative CT-scan showed bulging of the heart and lung parenchyma into the defect, but no signs of mediastinal infection. The entire surgical scar with the ulcerative defect was excised (Fig. 1). The dacron patch was completely removed, revealing a layer of fibrous scar tissue underneath the dacron patch. This tissue layer was considered to provide sufficient protection to the intra-thoracic organs, and the skin was closed in two layers over this layer of tissue. The patient was kept in the pediatric intensive care unit for one day. Patient developed fever after one day of operation which was managed with meropenem. Blood culture was negative and after 7 days of intravenous treatment, she was discharged.

**Figure:**
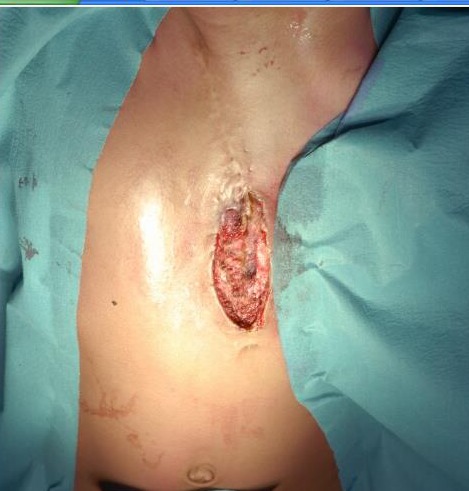
Figure 1:Infected scar at its lower half.

One month after surgery, she was again admitted with signs of a viral upper respiratory tract infection and mild wound dehiscence which was managed with wound dressings. After healing of the wound, a custom-made protective brace was applied. Outpatient follow-up included investigations into the underlying cause of the sternal defect. The sternal defect, micrognathia, high arched palate and small hemangiomas, in combination with the consanguinity of the parents were suggestive of some syndrome. An MRI of the brain showed no cerebral or vascular anomalies that correlated with PHACES syndrome. She was referred to the department of clinical genetics where no genetic cause could be found.

## DISCUSSION

Complete sternal cleft is rarely described in literature. About 24 cases of isolated sternal cleft are currently known.[1] It is thought that the abnormality is caused by an embryonic fusion defect of the mesenchymal plates during the eighth week of gestation.[2,3] Sternal agenesis can occur isolated or together with other congenital defects. PHACES syndrome is characterized by large facial hemangiomas. It also includes structural brain anomalies and arterial malformations causing neurologic symptoms, eye anomalies, cardiac and/or aortic disease, together with sternal defects. Only a part of the roughly 130 cases known in literature, describe sternal defects, which can be either partial or complete.[3] Our patient did have small facial hemangiomas, but did not have any of the other congenital anomalies associated with PHACES, future genetic testing could confirm a possible similar etiology. Another association of sternal cleft with hemangiomas and micrognathia is mentioned in literature. However, the patient described in the case-report only had a partial superior sternal defect, as well as extensive vascular anomalies and brain hemangiomas.[4]

The preferred treatment strategy in literature is adjoining the rudimental sternal bars in the neonatal period. During these first months of life, the compliant thorax wall tolerates the correction and provides optimal anatomical and cosmetic results. Bridging the defect with autogenous tissue or allogeneic materials is advised after neonatal period.[5] Our patient was initially managed with dacron patch which later on became colonized with multidrug-resistant Pseudomonas and resulted in recurrent wound infections and hypertrophied scar. We initially tried local treatment however on recurrence of wound infection, the dacron patch was removed. The reactive fibrous scar-tissue beneath the removed dacron patch was considered to provide enough stability to the thorax. She appeared to be more vulnerable to trauma, therefore, we applied a brace providing more protection to the intrathoracic organs.

## Footnotes

**Source of Support:** Nil

**Conflict of Interest:** None declared

